# Supplementary material for: Functional Characterization of Physcomitrella patens Glycerol-3-Phosphate Acyltransferase 9 and an Increase in Seed Oil Content in Arabidopsis by Its Ectopic Expression
Source: Plants (Basel). 2019 Aug 13;8(8):284. doi: 10.3390/plants8080284 (PMC6724121; doi:10.3390/plants8080284)
Supplement: Supplementary file 1 [file plants-08-00284-s001.pdf]

Supplementary Table S1. List of DNA primers used in this study.

Primer name	Sequence (5'-3')	Purpose
PpGPAT9 BamH1 F1	GGATCCTAATGGAAGGGGACCAGTTC	Overexpression of PpGPAT9
PpGPAT9 Spe1 R1	GGACTAGTTTACTGATTTTGAGCTGTTGC	
PpGPAT9 BamH1 F1	GGATCCTAATGGAAGGGGACCAGTTC	Subcellular localization
PpGPAT9 Sal1 R2	CCGTCGACTTACTGATTTTGAGCTGTTG	
PpGPAT9 RT F1	TAGGACAGAGGCCAATGACCG	qRT-PCR
PpGPAT9 RT R1	GCTGTTCCAGAAGGCATCCAC	
PpACT2 RT F1	GTATTTCTTCTCCATGGCTGG	qRT-PCR
PpACT2 RT R1	CCTCTTCGACTGAGCTTCGT	
